# Biomarkers to Improve Diagnosis and Monitoring of Obstructive Sleep Apnea Syndrome: Current Status and Future Perspectives

**DOI:** 10.1155/2014/930535

**Published:** 2014-11-27

**Authors:** Konstantinos Archontogeorgis, Evangelia Nena, Nikolaos Papanas, Paschalis Steiropoulos

**Affiliations:** ^1^Department of Pneumonology, Medical School, Democritus University of Thrace, 68100 Alexandroupolis, Greece; ^2^Laboratory of Hygiene and Environmental Protection, Medical School, Democritus University of Thrace, 68100 Alexandroupolis, Greece; ^3^Second Department of Internal Medicine, Medical School, Democritus University of Thrace, 68100 Alexandroupolis, Greece; ^4^Sleep Unit, Department of Pneumonology, Medical School, Democritus University of Thrace, 68100 Alexandroupolis, Greece

## Abstract

Obstructive sleep apnea syndrome (OSAS) is characterized by recurrent episodes of upper airway collapse associated with oxygen desaturation and sleep disruption. It is proposed that these periodic changes lead to molecular variations that can be detected by assessing serum biomarkers. Studies have identified inflammatory, oxidative, and metabolic perturbations attributable to sleep-disordered breathing. Given that OSAS is associated with increased cardiovascular and cerebrovascular morbidity, the ideal biomarker should enable timely recognition with the possibility of intervention. There is accumulating data on the utility of serum biomarkers for the evaluation of disease severity, prognosis, and response to treatment. However, current knowledge is limited by data collection techniques, disease complexity, and potential confounding factors. The current paper reviews the literature on the use of serum biomarkers in OSAS. It is concluded that the ideal serum biomarker still needs to be discovered, while caution is needed in the interpretation of hitherto available results.

## 1. Introduction

Obstructive sleep apnea syndrome (OSAS) is characterized by recurrent episodes of upper airway collapse associated with oxygen desaturation and sleep fragmentation [1]. It is a common disorder, with prevalence estimated at 4% for men and 2% for women among adults in the Western countries [2]. Considering the rising incidence of obesity, a recognized risk factor for OSAS, the prevalence of this condition will probably increase. OSAS is associated with increased risk for cardiovascular disease [3] and cerebrovascular morbidity [4].

Oxygen desaturation accompanying apneic events, negative intrathoracic pressure, arousals induced by upper airway obstruction, and repeated activation of the sympathetic system may cause abnormal activation of neural, humoural, thrombotic, metabolic, and inflammatory responses, thereby promoting atherosclerosis [5, 6]. The potential etiological mechanisms may include endothelial dysfunction, oxidative stress, inflammation, and disorders of coagulation and lipid metabolism [7].

OSAS is diagnosed by polysomnography (PSG) and is treated by Continuous Positive Airway Pressure (CPAP) application, oral appliances, surgery, and weight loss. However, it often remains undiagnosed and untreated. Indeed, recent investigations have shown that the probable reason for this underdiagnosis is lack of physicians' awareness [8]. Furthermore, patients are often reluctant to undergo overnight PSG and follow the appropriate therapy.

A biomarker is defined as a characteristic that is objectively measured and evaluated as an indicator of normal biologic processes, pathogenic processes, or pharmacologic response to a therapeutic intervention [9]. In the case of OSAS, the ideal biomarker should be useful for the following: diagnosis, assessing disease burden and severity, and evaluating response to treatment [10]. To ensure diagnostic accuracy, a biomarker should yield both high sensitivity and specificity. Such diagnostic performance would limit the need of PSG, an expensive and laborious modality, at least in some patients. For the evaluation of disease severity and response to treatment, a biomarker should be implicated in the pathogenesis of complications and should respond to therapy. Variations in biomarker levels as response to therapy would then reflect reduction of complications. Additional aspects should include low cost and facility in use, as well as the ability to simultaneously evaluate multiple pathogenic pathways.

The aim of this review is to summarize current knowledge on the utility of serum biomarkers in OSAS.

## 2. Search Strategy

We performed an electronic search in PubMed, EMBASE, and Google Scholar, using combinations of the following keywords: OSAS, biomarkers, serum, inflammation, oxidative stress, and metabolic. References from systematic reviews and meta-analyses were also screened for relevant studies. The search included all types of articles written in English up to April 2014. Cross-sectional, case control, and cohort studies with comparison group data were included in this review. Studies with historical controls, case series, and case reports were excluded and also studies published in any language other than English were excluded.

Regarding inflammatory markers a total of 229 studies were initially considered (69 for CRP, 43 for TNF-*α*, 16 for IL-8, 49 for IL-6, 25 for cell adhesion molecules, and 27 for fibrinogen) and 59 were finally included in this review (16 for CRP, 13 for TNF-*α*, 5 for IL-8, 11 for IL-6, 6 for cell adhesion molecules, and 8 for fibrinogen). A total of 42 studies were revised on the relationship between OSAS and oxidative stress (10 for antioxidant capacity, 8 for 8-isoprostane, 8 for malondialdehyde, 8 for total antioxidant status, and 8 for paraoxonase) and 18 of them were included in the present review (3 for antioxidant capacity, 4 for 8-isoprostane, 5 for total antioxidant status, and 6 for paraoxonase). Our search regarding metabolic markers included 117 studies (27 for hemoglobin A1c, 47 for leptin, 36 for adiponectin, and 7 for resistin) and 29 studies were finally included in the current review (11 for hemoglobin A1c, 6 for leptin, 9 for adiponectin, and 3 for resistin).

## 3. Inflammatory Markers

A variety of studies have implicated OSAS as an important risk factor in the development of cardiovascular disease [3]. Although it is postulated that sleep deprivation and hypoxemia may contribute to the etiology of cardiovascular events, the exact mechanisms need further elucidation [11, 12].

According to one hypothesis, OSAS-induced hypoxic stress may increase circulating levels of biochemical mediators of inflammation. On the other hand, there is increased prevalence of obesity among patients with OSAS, and previous evidence suggests that adipose tissue can produce proinflammatory factors, such as C-reactive protein (CRP), tumor necrosis factor-*α* (TNF-*α*), interleukin-6 (IL-6), and interleukin-8 [13–16].

### 3.1. CRP

CRP is a marker of inflammation that is mainly produced by the liver in response to IL-6. Increased CRP levels are identified as consequence of trauma or infection. Data suggests an active role in the atherogenic process mediated by the expression of adhesion molecules and monocyte activation [17]. Moreover, increased levels of CRP are recognized as an independent risk factor of future cardiovascular events among healthy subjects, as well as in patients with known cardiovascular disease [18, 19].

The relationship between OSAS and CRP has been the subject of various studies with contradictory results. Considerable evidence supports an independent association between serum CRP levels and OSAS [20]. In a comparison of 22 otherwise healthy OSAS patients with 20 subjects matched for age and body mass index (BMI) without OSAS, CRP levels were significantly higher among the former (0.33 versus 0.09 mg/dL, *P* < 0.0003), and they were independently associated with disease severity [21]. Similar results were reported by Hayashi et al. [22]. Among 60 patients, repetitive hypoxemia was significantly correlated with high sensitivity-CRP (hs-CRP) and appeared to be a fundamental determinant for this molecule. CRP levels were also correlated with indices of nocturnal hypoxia (average or minimum SpO_2_ during sleep), suggesting that hypoxia is probably the major contributor in the activation of inflammation in OSAS [23].

In another study on 76 patients, hs-CRP levels between a group of OSAS patients and a control group matched for age, sex, and BMI were compared. Hs-CRP levels were higher in the OSAS group in comparison to controls and were positively correlated with BMI (*r* = 0.376, *P* = 0.001) and apnea-hypopnea index (AHI) (*r* = 0.280, *P* = 0.014). Multiple regression analysis demonstrated an association of hs-CRP levels with AHI (*F* = 3.293, *P* = 0.033), which was independent of obesity [24].

In a comparison between 161 OSAS patients and 61 controls, serum CRP levels were significantly higher in the OSAS group (<1 mg/dL in controls versus 2 mg/dL in OSAS patients, *P* = 0.001), and AHI was an independent predictor for CRP. Even though OSAS patients had higher age and BMI than controls, the association between CRP levels and AHI remained significant after adjustment for these variables (*P* < 0.001), while this association was not significant when additional factors (cardiovascular disease, smoking, systolic blood pressure) were considered [25].

Yokoe et al. [26] have found that CRP levels in OSAS patients were higher when compared with obese control subjects and were decreased after 30 days of CPAP therapy. Serum levels of cardiovascular risk factors, including hs-CRP, were decreased after 6 months of CPAP application in a group of OSAS patients (*P* = 0.03) [27]. However, Kohler et al. [28] have found that 4 weeks of CPAP treatment had no beneficial effect on CRP levels in a group of patients with moderate-severe OSAS and comorbidities.

In a meta-analysis of 14 studies with a total of  771 patients, CRP levels ranged from 0.18 to 0.85 mg/dL before CPAP treatment and from 0.10 to 0.72 mg/dL after CPAP therapy [29]. The pooled mean difference was 0.14 (95% confidence interval (95% CI) 0.08–0.20, *P* < 0.00001) [29]. A further meta-analysis of 30 studies assessing the relationship between CRP and OSAS confirmed that CRP was higher in OSAS patients compared with controls (pooled mean difference 1.77) [30].

Conversely, several authors have failed to demonstrate a relationship between CRP levels and OSAS [31, 32]. In other cases, this association was not significant after adjustment for obesity [12, 23]. In an analysis of 156 subjects with OSAS [31], 39 subjects with upper airway resistance syndrome, and 54 controls, serum CRP levels were normal in all groups and only BMI was significantly associated with high CRP values (*P* < 0.001). Ryan et al. [32] have compared serum CRP levels in a group of non-OSAS subjects with those of a group of mild/moderate and a group of severe OSAS patients matched for BMI and with a group of more obese OSAS patients. CRP levels were similar among the BMI-matched groups, but they were significantly increased in the more obese group (*P* < 0.05). Multivariate analysis of all subjects demonstrated that BMI, but not AHI, was an independent predictor of CRP levels. In the same study, 6 weeks of CPAP treatment did not alter serum CRP levels. Similarly, in a cohort of 454 untreated OSAS patients, OSAS severity was an independent predictor of CRP levels but interacted with obesity and thus was found only in obese patients [33].

The finding of increased serum CRP levels in OSAS patients and its association with disease severity may reflect the risk of developing cardiovascular events or the presence of subclinical atherosclerosis. Therefore, therapeutic interventions that aim at decreasing serum CRP levels may have a protective role for future cardiovascular and cerebrovascular complications.

### 3.2. TNF-*α*

TNF-*α* is a proinflammatory cytokine produced by monocytes/macrophages during acute inflammation [34]. It participates in a wide spectrum of signaling events that lead to necrosis or apoptosis [34]. It is also involved in sleep regulation and has been positively correlated with excessive daytime sleepiness, nocturnal sleep disturbance, and hypoxia [35].

High serum levels of TNF-*α* have been shown to be produced by monocytes of OSAS patients [36]. Additionally, previous studies have demonstrated increased levels of TNF-*α* in such patients during the morning and immediately after the first episode of obstructive apnea. Several datasets have confirmed the relationship between increased serum TNF-*α* levels and OSAS [37, 38]. Steiropoulos et al. [23] found higher TNF-*α* levels in a group of OSAS patients compared to a group of BMI-matched control subjects (6.72 ± 3.72 versus 3.94 ± 1.34 pg/mL, *P* < 0.001) [23]. Serum TNF-*α* was positively correlated with neck circumference (*r* = 0.452, *P* < 0.001), AHI (*r* = 0.391, *P* = 0.002), and oxygen desaturation index (ODI) (*r* = 0.384, *P* = 0.002). Similar results were observed by Ciftci et al. [39].

In a comparison between 26 subjects with mild OSAS, 54 subjects with moderate/severe OSAS, and 40 controls, matched for age and BMI, TNF-*α* levels were significantly increased in the moderate/severe group, as compared with the other patients (22.83 ± 3.85 for moderate/severe OSAS versus 14.42 ± 3.29 for mild OSAS versus 12.53 ± 3.48 pg/mL for controls, *P* < 0.01) [40]. Serum TNF-*α* levels were significantly correlated with carotid intima-media thickness (*r* = 0.53, *P* < 0.01), an early marker of atherosclerosis [40].

In another work [36], serum TNF-*α* levels and its spontaneous production by monocytes were increased in OSAS patients, as compared with obese subjects (*P* < 0.0005 and *P* < 0.0001, resp.) and healthy individuals (*P* < 0.0001 and *P* < 0.0001, resp.) and were increased in parallel with disease severity. Interestingly, both spontaneous production and serum concentration of TNF-*α* were independently associated with duration of hypoxia during total sleep time, and they were decreased after one month of CPAP application [36]. Moreover, prior evidence demonstrated that increased levels of serum TNF-*α* can be attenuated after CPAP treatment [41, 42].

In a meta-analysis of pooled data from 9 studies with a total of 209 patients, mean TNF-*α* levels ranged from 1.40 to 50.24 pg/mL before CPAP treatment and 1.80 to 28.63 pg/mL after CPAP application. The pooled mean difference was 1.14 (95% CI 0.12–2.15, *P* = 0.03) [29]. Another meta-analysis of 19 studies concluded that TNF-*α* serum levels were increased in OSAS patients in comparison to nonapneic controls (pooled mean difference 1.03) [30].

However, there is also evidence that there may be no difference in serum TNF-*α* among OSAS patients compared with controls, before and after therapy with CPAP [43, 44]. Guasti et al. [44] compared TNF-*α* serum levels and production from peripheral blood mononuclear cells between 16 OSAS patients and 11 controls matched for cardiovascular risk factors, except obesity. Serum TNF-*α* was normal in both groups, and production from peripheral blood mononuclear cells was similar between groups. Treatment with CPAP for 12 weeks did not alter TNF-*α* serum levels [44]. Obviously, obesity may represent the primary confounding factor affecting the levels of TNF-*α* in these works. Indeed, macrophages of adipose tissue can also secrete TNF-*α* [45].

Even though there is a role for OSAS in promoting inflammation among obese individuals, possibly mediated by TNF-*α*, this interaction is quite complex and merits further study. This increased production of proinflammatory cytokines may affect patient outcomes, especially regarding increased frequency of cardiovascular and cerebrovascular complications associated with OSAS.

### 3.3. IL-8

IL-8 is a chemokine produced by macrophages and other cell types, such as epithelial cells, airway smooth muscle cells, and endothelial cells [46]. It has been demonstrated that hypoxia can positively affect the expression and production of this chemokine [47]. Previous reports have examined the expression of IL-8 in individuals with OSAS. Ohga et al. [48] have compared IL-8 levels of 20 OSAS patients before and after CPAP treatment and 10 control subjects matched for age and BMI. IL-8 levels in the OSAS group were significantly higher than those in the control group (*P* < 0.05) [48]. A significant correlation between IL-8 levels and desaturation magnitude was observed (*r* = 0.69, *P* < 0.001), while a positive correlation with apnea index was also suggested (*r* = 0.51, *P* = 0.004). CPAP treatment for at least 8 months decreased levels of this chemokine in the OSAS group [48].

Oyama et al. [49] have included 32 patients with OSAS and metabolic syndrome, finding that IL-8 serum levels were diminished after three months of CPAP treatment. In another study, IL-8 levels were increased in 25 severe OSAS patients when compared to 17 healthy individuals matched for age and BMI (198.8 ± 4.76 versus 180.83 ± 3.38 pg/mL, resp., *P* < 0.005) [50].

However, negative findings have been reported as well [44]. In a comparison of 16 OSAS subjects with 11 controls, both IL-8 serum levels and its release from neutrophils were similar between both groups [44]. Moreover, 12 weeks of CPAP therapy did not influence serum concentration of cytokines [44]. Additionally, an enquiry into the effects of intermittent hypoxia in healthy subjects found no increased levels in IL-8 [51]. The authors concluded that a more substantial or a different pattern of hypoxia may be necessary for the initiation of systemic inflammation [51]. Alternatively, any increase in serum markers of inflammation in OSAS could be more related to other factors, such as obesity or night-time arousals [51].

In summary, hypoxic events induced by OSAS increase serum levels of IL-8, a risk factor of cardiovascular disorders. By reducing this chemokine, CPAP might represent an efficacious way of reducing cardiovascular complications associated with OSAS.

### 3.4. IL-6

IL-6 is a circulating cytokine produced by a variety of cells, including macrophages and lymphocytes [52]. It is a major initiator of the acute phase response and the principal regulator of the hepatic CRP production. Available data on IL-6 levels are often conflicting. Indeed, some groups have reported increased levels in OSAS, while others have demonstrated that obesity* per se* rather than OSAS was associated with systemic inflammation [23, 26, 39, 41, 43]. Specifically, IL-6 levels are higher in obesity, and their production is 2-3 times higher in visceral fat compared with subcutaneous fat [53, 54]. Additionally, 15–30% of the circulating levels come from fat tissue [54].

Liu et al. [38] compared IL-6 levels in 22 OSAS patients and 16 normal controls. Plasma IL-6 levels of OSAS patients were significantly higher in comparison with controls (50.67 ± 4.70 versus 12.69 ± 2.75 pg/mL, resp.) [38]. A positive correlation was observed between the cytokine and percentage of time of apnea and hypopnea, as well as percentage of time with oxygen saturation below 90% [38]. However, there was a significant difference in BMI among groups (27.58 ± 3.28 for OSAS versus 23.11 ± 2.96 kg/m² for controls, *P* < 0.01). Maeder et al. [55] found higher IL-6 serum levels after sleep in patients with moderate/severe OSAS when compared with mild or no OSAS patients (*P* = 0.005), but not before sleep (*P* = 0.08).

In a cross-sectional cohort study [33] enrolling 454 untreated OSAS patients, IL-6 serum levels were significantly correlated with ODI, hypoxia time, and minimum oxygen saturation in sleep. Nevertheless, after stratification by BMI, disease severity was associated with IL-6 levels only in obese individuals (BMI > 30 kg/m²). Another group has included 23 healthy controls and 38 otherwise healthy OSAS patients and found no difference in serum IL-6 levels between the groups (2.36 ± 1.41 versus 2.73 ± 1.14 pg/mL, *P* = 0.465) [23].

In a meta-analysis of 18 studies, IL-6 plasma levels were increased among OSAS patients compared with controls (pooled mean difference 2.16) [30]. In a further meta-analysis of 8 studies [29] with a total of 165 patients, IL-6 level means ranged from 1.2 to 131.66 pg/mL before CPAP and from 0.45 to 66.04 pg/mL after CPAP. The pooled mean difference was 1.01 (95% CI 0.00 to 2.03, *P* = 0.05) [29]. Among the works included in this meta-analysis, only 3 out of 8 showed attenuated IL-6 serum levels after treatment, while there was no significant change in the remaining five [29]. Populations in these studies were small and exhibited comorbidities, like obesity.

Increased IL-6 levels are associated with OSAS severity and are often correlated with higher serum CRP levels, both cytokines being involved in the pathogenesis of cardiovascular and cerebrovascular disease.

## 4. Cell Adhesion Molecules

Adhesion of circulating leukocytes on the endothelial cells is considered as an important step in atherosclerosis initiation [56]. This process is enhanced by cellular adhesion molecules in response to proinflammatory cytokines, such as TNF-*α* and interleukin-1 [57]. Potential mediators in the leucocyte attachment to endothelium process include intercellular adhesion molecule-1 (ICAM-1), vascular cell adhesion molecule-1 (VCAM-1), and L-selectin [58]. The presence of these molecules is required for leucocyte migration in an inflamed area [59]. Previous reports have shown that hypoxia induces the expression of adhesion molecules in various cells, such as leukocytes and endothelial cells, also increasing neutrophil adherence to endothelium [60]. Although the role of cell adhesion molecules in OSAS has not yet been completely elucidated, there is evidence of elevation of circulating molecules [61–63].

In a study comparing 39 patients with moderate-to-severe OSAS with 34 nonapneic controls matched for age, gender, and BMI [64], circulating levels of both ICAM-1 and VCAM-1 were significantly higher in the OSAS group, as compared with the control group (480.1 ± 216.7 versus 303.4 ± 98.6 ng/mL, *P* < 0.0001, and 1156.6 ± 79.8 versus 878.8 ± 71.1 ng/mL, *P* = 0.002, resp.). Additionally, a significant positive correlation of ICAM-1 levels and AHI was observed (*r* = 0.276, *P* = 0.018) [64]. Multiple logistic regression analysis showed that the association between ICAM-1, VCAM-1 levels and OSAS was independent of age, gender, BMI, smoking status, and cardiovascular disease [64].

Ohga et al. [65] have found increased circulating levels of ICAM-1, VCAM-1, and L-selectin in OSAS patients before sleep, compared with a group of normal individuals matched for age, gender, and BMI (ICAM-1: 392.9 ± 48.5 versus 201.2 ± 55 ng/mL, *P* < 0.05; VCAM-1: 811.0 ± 87.8 versus 574.2 ± 42.7 ng/mL, *P* < 0.05; L-selectin: 1386.6 ± 77.9 versus 1038.8 ± 78.6 ng/mL, *P* < 0.01, resp.). However, after sleep, only levels of L-selectin and ICAM-1 were significantly increased in OSAS subjects, suggesting that VCAM-1 levels might not be directly related to hypoxia-induced inflammation [65]. Similarly, in another report, increased levels of adhesion molecules were correlated with AHI and ODI but not with indices of hypoxemia severity and frequency of arousals [66].

A meta-analysis of 8 studies for ICAM, six studies for VCAM, and five studies for selectins has demonstrated higher serum levels of all molecules in OSAS patients compared with controls (pooled mean difference 2.93 for ICAM, 2.08 for VCAM, and 1.45 for selectins) [30]. Furthermore, a significant decrease in serum levels of cellular adhesion molecules was reported after one month of CPAP treatment [67]. Although the abovementioned reports illustrate the importance of these markers, they mostly include studies with a limited number of patients in which serum concentrations were measured by a single blood sample. Due to these limitations, it is conceivable that they may not accurately reflect their action at the level of vascular endothelium [68].

Taken together, results from various studies suggest that OSAS-induced hypoxia activates adhesion molecules. These inflammatory markers are associated with worse cardiovascular prognosis. Importantly, CPAP therapy could improve cardiovascular outcomes by a beneficial reduction of these markers.

## 5. Fibrinogen

Fibrinogen is an acute phase protein formed in the liver, and it may be elevated in various types of inflammation. Increased plasma levels of fibrinogen have been identified as a major independent risk factor for cardiovascular and cerebrovascular events [69]. Fibrinogen levels were increased in patients with coexisting OSAS and stroke, indicating a possible pathophysiological mechanism for the increased risk of stroke in such patients [70]. Hypoxia enhances fibrinogen production, and, indeed, morning levels of fibrinogen were found increased in OSAS patients compared with nonapneic controls [71, 72]. The morning increase of fibrinogen concentration was attenuated after CPAP treatment [73].

The role of fibrinogen in OSAS has been widely studied [74, 75]. In a recent investigation by Shamsuzzaman et al. [76], plasma fibrinogen levels were increased in patients with severe OSAS compared with mild OSAS patients and controls matched for age, BMI, smoking habits, and hemodynamics (398 ± 18 versus 331 ± 25, *P* = 0.003 and 398 ± 18 versus 334 ± 25 mg/dL, *P* = 0.02, resp.). However, no significant difference was noted in fibrinogen levels between mild OSAS group and controls, suggesting that the partial sleep loss due to arousals and the level of hypoxia during sleep may represent the main regulators of fibrinogen levels [76]. Serum fibrinogen levels were directly related to AHI and arousal index and inversely related to mean and lowest oxyhemoglobin saturation during sleep [76].

An analysis of 10 studies on hemostatic variables in OSAS has demonstrated that the average minimal oxygen saturation was an independent predictor of fibrinogen [77]. However, a number of authors failed to demonstrate an association between plasma fibrinogen levels and OSAS [23, 78]. In a study including 30 children with snoring and OSAS, 61 children with snoring and no OSAS, and 23 controls, fibrinogen levels were higher in the OSAS group compared with both non-OSAS snorers and control groups (318 versus 307 and 271 mg/dL, resp., *P* < 0.05) [78]. Factors influencing serum fibrinogen levels commonly coexisting in OSAS include obesity, age, tobacco smoking, and alcohol consumption [74, 79].

In summary, serum fibrinogen levels are increased in OSAS patients and are associated with disease severity, indicating increased risk for thrombosis.

## 6. Markers of Oxidative Stress

Oxidative stress is characterized by an imbalance between oxidant mechanisms, such as reactive oxygen species, and the antioxidant system that includes various enzymatic and nonenzymatic processes. Reactive oxygen species react with DNA, lipids, and proteins, leading to cell injury, endothelial damage, and vasoconstriction, thereby increasing the risk of atherosclerosis [80]. The presence of systemic oxidative stress is considered as an important mechanism linking OSAS with cardiovascular disease and metabolic perturbations [81]. Repetitive episodes of upper airway collapse followed by adequate ventilation cause multiple periods of hypoxia and reoxygenation. These repeated events are considered to play a role analogous to hypoxia-reperfusion injury, which enhances oxidative stress [82].

Previous data have indicated increased oxidative stress in OSAS patients along with decreased antioxidant capacity compared with healthy controls [83]. Conversely, several reports failed to demonstrate such an association, possibly due to confounding factors [84, 85]. Lee et al. [84] have compared serum total antioxidant status between a group of 31 mild-to-moderate OSAS patients, a group of 22 severe OSAS patients, and 20 controls matched for age, BMI, but not smoking habits. Total antioxidant status was similar among groups considered (1.87 ± 0.3 for mild/moderate OSAS, 1.94 ± 0.32 for severe OSAS, and 1.96 ± 0.32 mmol/L for controls, resp., *P* = 0.53).

Isoprostanes are a class of prostanoids formed by free radical-catalyzed peroxidation of essential fatty acids, primarily arachidonic acids [86]. In the study of Tan et al. [87], plasma 8-isoprostane levels were elevated in a group of 128 OSAS patients compared with 82 controls (125.8 [94.9–256.2] versus 120.6 [86.4–166.3] pg/mL resp., *P* < 0.01). However, OSAS patients were older and exhibited higher BMI than controls [87]. Relevant conclusions were reached by Barceló et al. [88], who compared serum 8-isoprostane levels of 119 OSAS patients and an equal number of controls matched for sex, age, and BMI (11.4 versus 4.3 U/L, resp., *P* = 0.001). In a randomized crossover study by Fernández et al. [89] assessing the effect of CPAP therapy on oxidative stress, plasma 8-isoprostane levels were significantly decreased after 12 weeks of treatment (38.5 [24.2–58.7] at baseline versus 22.5 [16.2–35.3] pg/mL after treatment, *P* = 0.0001) in 31 newly diagnosed OSAS patients without comorbidities.

However, another group has measured serum 8-isoprostane levels in 41 moderate-severe OSAS patients and 35 matched controls before sleep, after 4 hours of untreated OSA and after 4 hours of effective treatment with CPAP [90]. 8-isoprostane levels in OSAS patients were comparable to those of control individuals and remained unaffected by CPAP treatment and normal sleep period [90].

Malondialdehyde (MDA) is a product of lipid peroxidation, which may have implications in the development of cardiovascular disease [91]. Higher MDA levels are considered an index of increased stress-induced lipid peroxidation [92]. Several reports used MDA plasma levels as a parameter of oxidative stress in OSAS patients [93, 94]. Jurado-Gámez et al. [95] have included 46 OSAS patients and matched for age, sex, and BMI 23 non-OSAS subjects. They have found that MDA serum levels were increased among the former (*P* = 0.001) [95]. In the same work, serum levels were attenuated after 3 months of CPAP treatment (3.2 before and 1.9 *μ*M after CPAP therapy, *P* = 0.001). Similar findings were reported in another study [96]. In another study, MDA concentrations correlated with the duration of nocturnal desaturation below 85% (*r* = 0.77, *P* < 0.0005) [97]. At the same time, serum MDA levels were progressively increased during the night and they reached their maximum concentration one hour after awakening [97]. These observations are consistent with the hypothesis that intermittent hypoxia induces oxidative stress in OSAS patients.

Total levels of antioxidant enzymes, as well as total antioxidant status (TAS), have been studied in OSAS patients. Cofta et al. [98] have found that TAS serum concentration was progressively decreased in comparison with matched control subjects and OSAS patients of increasing severity (1.58 for controls, 1.34 for AHI 5–15, 1.46 for AHI 16–30, and 1.38 mmol/L for AHI ≥ 30, *P* = 0.03). Similar results were reported by other authors [99]. TAS values were found lower immediately after sleep, compared with those before sleep, in individuals with less severe OSAS but not in those with severe syndrome (1.73 ± 0.08 versus 1.65 ± 0.09 mmol/L, *P* = 0.01; and 1.64 ± 0.12 versus 1.58 ± 0.10 mmol/L, *P* = 0.07, resp.) [83]. These results may reflect the difference of the acute effect of hypoxemia induced by apneic sleep and a chronic state of oxidative stress that may exist in severe syndrome even during daytime. TAS levels were negatively correlated with arousals (*r* = −0.29, *P* = 0.04) [100].

However, a recent study failed to demonstrate the abovementioned relationships [101]. Total antioxidant capacity was measured in 18 patients with severe OSAS and 13 control subjects before and the morning after PSG [101]. Results showed no difference in total antioxidant capacity between groups (0.92 ± 0.16 versus 0.98 ± 0.19 m MDPPH, resp., *P* = 0.41).

Paraoxonase (PON) is an antioxidant enzyme protecting low-density lipoprotein from oxidation [102]. Lavie et al. [103] have reported higher serum levels of lipid peroxidation products and lower PON-1 levels in OSAS patients with and without cardiovascular disease compared with controls (79.5 ± 13.6 versus 86.7 ± 17.6 versus 92.1 ± 14.4 U*·*min/mL, resp., *P* = 0.002). They have also observed a negative correlation between PON-1 activity and RDI (*r* = −0.24, *P* < 0.01) [103]. Other works have also confirmed decreased plasma levels of PON in OSAS compared with controls [99, 104].

In another study, PON-1 activity was lower in OSAS patients and in COPD patients compared with controls, suggesting that both nocturnal intermittent and sustained hypoxemia may have an effect on diminished PON-1 concentration [105]. CPAP treatment has proven to increase levels of PON-1 in OSAS patients (104.0 ± 10.5 before treatment versus 120.3 ± 8.6 U/L after treatment) [106]. By contrast, Vatansever et al. [94] showed no significant difference in PON activity between OSAS patients and controls. The authors suggested that PON activity alterations probably depend on the presence of cardiovascular disease in OSAS [94].

In conclusion, OSAS is associated with increased oxidative stress. Putative underlying mechanisms include increased production of oxidant factors or decreased antioxidant capacity, both due to hypoxia induced by apneic events. Further studies with adequately selected patients are needed to clarify the role of OSAS in inducing an oxidative state.

## 7. Metabolic Markers

Evidence suggests that intermittent hypoxia and oxyhemoglobin desaturation, both hallmarks of OSAS, may induce impaired glucose and lipid metabolism, as well as insulin resistance and metabolic syndrome [107, 108]. Hemoglobin A1c (HbA_1c_) has been found elevated in nondiabetic OSAS patients and was negatively correlated with minimum oxyhemoglobin desaturation levels (*r* = −0.302, *P* = 0.018) and positively correlated with mean desaturation index (*r* = 0.263, *P* = 0.041) [109]. In a cross-sectional observational study on obese patients, a significant U-shaped association (*R* ^2^ = 0.241, *P* < 0.0005) between sleep duration and HbA_1c_ was found [110]. Decreased duration of slow-wave sleep (N3) was associated with decreased insulin secretion [110]. The relationship between OSAS and HbA_1c_ has been extensively investigated [111, 112].

Priou et al. [113] investigated the association between OSAS severity and HbA_1c_ in 1599 patients without diabetes. A positive association between HbA_1c_ and severity of OSAS was observed (5.6 ± 0.3% for AHI <5, 5.6 ± 0.3% for 5 ≤ AHI ≤ 15, 5.7 ± 0.3% for 15 ≤ AHI ≤ 30, 5.7 ± 0.4% for 30 ≤ AHI ≤ 50, and 5.8 ± 0.3% for AHI ≥ 50, *P* < 0.0001). Additionally, a dose-response relationship was demonstrated between AHI and percentage of patients with HbA_1c_ > 6.0% ranging from 10.8% for AHI < 5 to 34.2% for AHI ≥ 50, suggesting an increased risk for developing diabetes among these patients. In nondiabetic subjects, HbA_1c_ was positively correlated with arousal index (*β* = 0.448, *P* = 0.014) and percentage of sleep time with oxyhemoglobin saturation below 90% (*β* = 0.470, *P* = 0.01) and negatively with average oxyhemoglobin saturation (*β* = −0.456, *P* = 0.012) [114].

The effect of CPAP therapy on glycemic control has been the subject of many investigations, often leading to conflicting results [115, 116]. Results from two meta-analyses showed no significant influence of CPAP treatment on HbA_1c_, while another meta-analysis showed an improvement of insulin resistance in nondiabetic OSAS subjects [117–119]. Further studies are needed in order to evaluate the effects of long time therapy of OSAS on glycemic control.

Leptin is secreted by adipocytes in proportion to fat mass, and it suppresses appetite [120]. Leptin levels have been evaluated in OSAS patients [62, 121]. Increased leptin levels have been found in patients with severe OSAS compared with controls (9.05 ± 5.91 versus 4.24 ± 1.78 ng/mL, *P* < 0.01) [122]. Serum leptin levels were positively correlated with AHI (*r* = 0.552, *P* < 0.001) and oxyhemoglobin saturation below 90% (*r* = 0.399, *P* < 0.001), as well as BMI (*r* = 0.807, *P* < 0.0001), demonstrating an increase of leptin levels with increasing OSAS severity [122]. However, there was a significant difference in BMI between OSAS patients and controls, which may be relevant [122].

By contrast, several authors have failed to demonstrate an association between leptin levels and OSAS [123, 124]. A study comparing leptin levels between obese and nonobese OSAS patients and obese and nonobese controls found no differences in serum concentrations between these groups (19.27 for obese OSAS versus 16.06 ng/mL for obese controls, *P* = 0.848; and 7.02 for nonobese OSAS versus 7.85 ng/mL for nonobese controls, *P* = 0.393). Leptin levels differed significantly between obese and nonobese OSAS patients (19.27 versus 7.02 ng/mL, *P* < 0.001) and obese and nonobese controls (16.06 versus 7.85 ng/mL, *P* = 0.004). After 3 months of CPAP treatment, leptin levels were significantly decreased only in obese OSAS patients (19.27 before and 15.48 ng/mL after treatment, respectively, *P* = 0.016) [124].

Adiponectin is secreted by adipose tissue and is inversely related to the percentage of body fat, also exhibiting a negative correlation with insulin resistance [125]. Its plasma levels in OSAS patients were negatively correlated with sympathetic activation, expressed as urinary secretion of norepinephrine and normetanephrine (*r* = −0.231, *P* < 0.008), possibly resulting from sleep-disordered breathing [126]. Additional evidence suggests that hypoxic stress may reduce serum adiponectin in OSAS patients by acting at transcriptional and posttranscriptional levels [127]. Zhang et al. [128] found significantly lower adiponectin levels in OSAS patients compared with simple snorers (4.56 ± 2.12 versus 7.66 ± 3.71 *μ*g/mL, resp., *P* < 0.01). Of note, this difference was more pronounced in the group of moderate and severe syndrome, thus suggesting an independent effect of disease severity on adiponectin serum levels [128]. Serum adiponectin levels were also negatively correlated with AHI (*r* = −0.361, *P* < 0.01), but positively correlated with nadir oxyhemoglobin saturation (*r* = 0.306, *P* < 0.05) [128].

In another study [37], plasma adiponectin levels were negatively correlated with time spent in oxyhemoglobin saturation below 90% (*r* = −0.229, *P* = 0.007) and positively with minimum oxygen saturation (*r* = 0.250, *P* = 0.003). However, the robust relationship of adiponectin levels with body fat and the presence of obesity has led various studies to conflicting results, with some demonstrating an association between OSAS and low adiponectin levels and others emphasizing the role of obesity as an important confounding factor [94, 122, 129]. Tokuda et al. [122] have compared serum adiponectin levels between 21 moderate OSAS patients, 32 severe OSAS patients, and 15 controls. There was no difference in serum adiponectin levels among the groups (4.28 ± 1.92 for moderate OSAS versus 4.58 ± 2.47 for severe OSAS versus 4.61 ± 1.81 *μ*g/mL for controls). Serum adiponectin levels were the determinant factor for %T < 90. Similar controversy also exists regarding the effects of CPAP therapy [28, 123, 130–132].

Resistin is an adipocytokine implicated in the development of obesity, insulin resistance, and metabolic syndrome [133, 134]. In a study comparing 31 OSAS patients with 10 matched controls, higher resistin plasma levels were found in OSAS (22.0 versus 15.8 ng/mL, resp., *P* < 0.05) with an increasing trend with progressing disease severity (*P* < 0.05) [135]. The average oxyhemoglobin saturation during sleep was the principal determinant of resistin levels (*P* < 0.01), while CPAP therapy decreased serum levels to baseline [135]. Conversely, other groups have shown weak or no correlation between OSAS and resistin levels, indicating body weight as the dominant modulator of insulin resistance [123, 136]. In a study including 20 obese OSAS patients, 6 months of CPAP treatment did not affect resistin serum levels (3.1 ± 0.4 before treatment versus 3.2 ± 0.4 ng/mL after treatment, *P* = 0.79) [123].

Diabetes mellitus represents a frequent comorbidity among OSAS patients and may further increase the risk of cardiovascular complications [137]. Even though studies investigating the effects of CPAP treatment on glycemic control brought conflicting results, the evaluation of serum biomarkers may help to distinguish a group of OSAS patients that may benefit from some therapeutic intervention.

## 8. New Biomarkers

Ongoing research has seen the identification of novel biomarkers of OSAS. Additional studies on a larger scale are necessary to investigate the utility of these markers in OSAS including treatment effect. Matrix metalloproteinases (MMPs) are endoproteases that cleave extracellular matrix and play an important role in cardiac and vascular remodeling [138]. Production of MMPs is stimulated by hypoxia and by various cytokines [139]. Monocytes isolated from OSAS patients appear to produce more MMP-9 than those from controls [139, 140]. Variability in serum level of MMPs among OSAS patients is also under investigation [140].

Tazaki et al. [141] have measured serum levels and enzymatic activity of MMP-9 in OSAS patients and a group of matched controls. A significant increase was observed among the former (91.9 ± 9.8 for controls versus 120.3 ± 7.8 for mild OSAS, *P* < 0.05, and 168.0 ± 18.9 ng/mL for moderate to severe OSAS, *P* < 0.01). Enzymatic activity was also increased in OSAS patients [141]. OSAS severity emerged as the primary factor influencing MMP-9 levels and activity, while one month of CPAP treatment significantly decreased both parameters [141]. Impressively, in OSAS patients sustaining uvulopalatal flap surgery, postoperative serum concentrations of MMP-9 were significantly lower in comparison to preoperative values [142].

Thioredoxin (TRX) is a protein playing a protective role against oxidant injury [143]. In animal models, increased tissue TRX activity may be identified as a response to chronic intermittent hypoxia [144]. TRX was also highly expressed in OSAS tonsils at the mRNA and protein levels [145]. Serum TRX levels were increased in OSAS patients in comparison with healthy controls and were decreased after CPAP therapy [146]. Guo et al. [147] have found that TRX plasma levels were positively correlated to AHI (*r* = 0.313, *P* < 0.05) and negatively to the lowest oxygen saturation (*r* = −0.266, *P* = 0.037). A cut-off value of 9.39 ng/mL could identify OSAS patients (sensitivity 91%, specificity 78%), while the cut-off value that differentiated mild from moderate-severe syndrome was 11.79 ng/mL (sensitivity 75%, specificity 65%), suggesting a potential role for TRX levels as a measure of OSAS severity [147].

Cysteine is derived from homocysteine through a transsulfuration pathway and its increased plasma levels have been associated with cardiovascular risk [148]. Serum cysteine levels have already been found significantly higher in OSAS patients compared with non-OSAS controls (490.16 ± 67 versus 439.81 ± 76.12 mmol/L, resp., *P* < 0.01) and this difference was maintained after inclusion of nonobese patients [149]. Six-month CPAP treatment significantly decreased cysteine levels [149].

Finally, hepcidin is produced by the liver and its levels are increased in inflammation, mainly as a response to elevated IL-6 levels. Given the systemic inflammatory state and the increase of IL-6 in OSAS, hepcidin might emerge as a new biomarker in OSAS [150].

## 9. Concluding Comments

A large body of evidence points to an increased risk of cardiovascular disease, insulin resistance, and metabolic syndrome in OSAS [151]. As with any disease process, timely diagnosis and therapy may not only improve outcomes, but also reduce the likelihood of future complications. Even though studies have focused on various biomarkers that could facilitate OSAS diagnosis and management, an ideal marker has yet to be discovered (Table 1).

The pathogenesis of OSAS complications is multifactorial, including systemic inflammation, oxidative stress, and metabolic perturbations. A biomarker sensitive enough to specific aspects of OSAS may permit the design of interventions intended to modulate pathogenic pathways. The essence of treatment is maintaining airway patency during sleep through CPAP, oral appliances, surgery, and weight loss. Although considerable progress has been made, results are not always certain and long term follow-up is usually required (Table 2). Thus, the utility of new biomarkers might be important.

Use of overnight PSG for patient follow-up is the gold standard, but it may be criticized for being time consuming, not always widely available and for increasing cost. Therefore, biomarkers to evaluate treatment effect are highly desirable, given that they may contribute to reduced waiting time in sleep laboratories and insurance costs. Finally, sophisticated molecular techniques may help identify the ideal biomarker of the future [152]. For the time being, however, caution is needed in the interpretation of available results.

## Figures and Tables

**Table 1:** Studies assessing inflammatory biomarkers in obstructive sleep apnea syndrome.

Study	Biomarkers	Comparison groups	Comments
Sahlman et al. [20]	CRP TNF-*α* IL-6	84 mild OSAS patients versus 40 non-OSAS controls	CRP and TNF-*α*, but not IL-6, levels were higher in the OSAS group. CRP levels increased with the increase of AHI

Shamsuzzaman et al. [21]	CRP	22 OSAS patients versus 20 non-OSAS controls	CRP levels were higher in OSAS group and were independently associated with OSAS severity

Hayashi et al. [22]	CRP	60 OSAS patients versus 30 non-OSAS controls	CRP levels were higher in the OSAS group and increased with OSAS severity. Repetitive hypoxaemia correlated with CRP levels

Steiropoulos et al. [23]	CRP TNF-*α* IL-6 Fibrinogen	38 OSAS patients versus 23 non-OSAS controls	TNF-*α* levels were higher in OSAS patients and were correlated with neck circumference, AHI, and ODI. No differences in CRP, IL-6, and fibrinogen levels were recorded between groups

Guven et al. [24]	CRP	47 OSAS patients versus 29 non-OSAS controls	CRP levels were higher in the OSAS group and were positively correlated with BMI and AHI

Hall et al. [25]	CRP	161 OSAS patients versus 61 controls	CRP levels were higher in the OSAS group and were associated with AHI

Yokoe et al. [26]	CRP IL-6	30 OSAS patients versus 14 non-OSAS obese controls	CRP and IL-6 levels were higher in patients with OSAS. CRP levels were influenced by severity of OSAS and BMI. IL-6 levels were influenced by nocturnal hypoxia and BMI

Guilleminault et al. [31]	CRP	146 OSAS patients versus 39 UARS patients versus 54 non-OSAS controls	CRP levels did not differ among groups. BMI was associated with increased CRP values

Ryan et al. [32]	CRP	35 mild-to-moderate OSAS patients versus 31 severe OSAS patients versus 14 obese severe OSAS patients versus 30 non-OSAS controls	CRP levels were higher in the obese severe OSAS group than in the other groups. BMI was an independent predictor for CRP levels

Vgontzas et al. [35]	TNF-*α* IL-6	14 OSAS patients versus 11 obese non-OSAS controls versus 12 normal weight controls	TNF-*α* and IL-6 values were highest in the OSAS group and lowest in the lean controls. Their values for obese controls were between those of the other groups

Minoguchi et al. [36]	TNF-*α*	24 mild and moderate-to-severe OSAS patients versus 15 obese non-OSAS controls versus 12 healthy subjects	TNF-*α* levels were higher in patients with moderate-to-severe OSAS than in patients with mild OSAS, obese control subjects, or healthy subjects. The duration of hypoxia was independently associated with production of TNF-*α* by monocytes

Kanbay et al. [37]	TNF-*α*	106 OSAS patients versus 32 non-OSAS controls	TNF-*α* levels were increased in OSAS group, positively correlated with SaO_2_ <90% and BMI and negatively with adiponectin levels

Liu et al. [38]	TNF-*α* IL-6	22 OSAS patients versus 16 non-OSAS controls	TNF-*α* and IL-6 levels were higher in OSAS group and were correlated with percentage of time of apneas and hypopneas and with percentage of time spending at SaO_2_ <90%

Ciftci et al. [39]	TNF-*α* IL-6	43 obese OSAS patients versus 22 BMI matched non-OSAS controls	TNF-*α* and IL-6 levels were higher in OSAS group and were correlated with AHI but not with BMI

Ciccone et al. [40]	CRP TNF-*α* IL-6	26 mild OSAS versus 54 moderate-to-severe OSAS versus 40 controls	CRP, TNF-*α*, and IL-6 levels were higher in moderate/severe OSAS than in other groups

Imagawa et al. [43]	TNF-*α* IL-6	110 severe OSAS patients versus 45 non-OSAS controls	TNF-*α* and IL-6 levels did not differ between groups. BMI was correlated with the severity of the AHI

Guasti et al. [44]	TNF-*α* IL-8	16 OSAS patients versus 11 non-OSAS controls	Resting and stimulated TNF-*α* and IL-8 release, as well as serum levels, were similar in the two groups

Ohga et al. [48]	IL-8 ICAM-1	20 OSAS patients versus 10 non-OSAS controls	IL-8 and ICAM-1 levels were higher in OSAS patients and were correlated with apnea index and desaturation magnitude

Alzoghaibi [50]	IL-8	25 severe OSAS patients versus 17 non-OSAS controls	IL-8 levels were higher in OSAS group

Cofta et al. [61]	CRP E-selectin L-selectin P-selectin	60 OSAS patients versus 20 non-OSAS controls	CRP and selectins levels progressively increased with increasing severity of OSAS

Testelmans et al. [62]	CRP TNF-*α* IL-8 IL-6 ICAM-1	15 non-OSAS controls versus 12 patients with cardiovascular disease versus 15 OSAS patients with cardiovascular disease versus 15 non-obese OSAS patients versus 15 obese OSAS patients	CRP, TNF-*α*, IL-8, IL-6, and ICAM-1 levels were higher in patients with acute cardiovascular events and OSAS and were associated with AHI

Ursavas et al. [64]	ICAM-1 VCAM-1	39 moderate-to-severe OSAS patients versus 34 non-OSAS controls	ICAM-1 and VCAM-1 levels were increased in the OSAS group. ICAM-1 levels were correlated with AHI

Ohga et al. [65]	ICAM-1 VCAM-1 L-selectin	7 OSAS patients versus 6 non-OSAS controls	ICAM-1, VCAM-1, and L-selectin levels were increased in OSAS group before sleep compared with controls. Only ICAM-1 and L-selectin levels were increased after sleep

Basoglu et al. [74]	CRP Fibrinogen	36 obese OSAS patients versus 34 obese non-OSAS controls	CRP and fibrinogen levels were higher in obese OSAS patients

Shamsuzzaman et al. [76]	Fibrinogen	10 mild OSAS patients versus 26 severe OSAS patients versus 18 non-OSAS controls	Fibrinogen levels were elevated in patients with severe OSAS compared with other groups

**Table 2:** Effects of continuous positive airway pressure treatment on several biomarkers in obstructive sleep apnea syndrome.

Study	Biomarkers	CPAP treatment duration	Comments
Yokoe et al. [26]	CRP IL-6	1 month	CRP, IL-6 levels, and spontaneous IL-6 production by monocytes were significantly decreased after treatment

Steiropoulos et al. [27]	CRP	6 months	CRP levels were decreased in the group with good adherence to treatment

Kohler et al. [28]	CRP IL-6	1 month	CRP and IL-6 levels were not decreased after treatment

Minoguchi et al. [36]	TNF-*α*	1 month	Serum levels and spontaneous production of TNF-*α* were decreased in patients with moderate-to-severe OSAS

Steiropoulos et al. [41]	TNF-*α* IL-6	6 months	TNF-*α* levels were decreased in the good compliance group after treatment. IL-6 levels did not alter with treatment

Hegglin et al. [42]	TNF-*α*	8 months	TNF-*α* levels were decreased in men independently from compliance to CPAP use, but not in women

Guasti et al [44]	TNF-*α* IL-8	12 weeks	TNF-*α* and IL-8 levels remained unchanged after treatment

Ohga et al. [48]	IL-8 ICAM-1	8–18 months	IL-8 and ICAM-1 levels were decreased after treatment

Oyama et al. [49]	TNF-*α* IL-8 IL-6	3 months	TNF-*α*, IL-8, and IL-6 levels were decreased after treatment

Chin et al. [67]	VCAM-1 ICAM-1 E-selectin	3-4 days, 1 month, 6 months	After 1 month E-selectin and ICAM-1 levels but not VCAM-1 had decreased. After 6 months VCAM-1 levels did not change, while ICAM-1 levels further decreased
